# Dissecting the causal relationship between neuroticism and osteoarthritis: a univariable and multivariable Mendelian randomization study

**DOI:** 10.3389/fpsyt.2024.1333528

**Published:** 2024-03-08

**Authors:** Shuren Zhang, Junhui Ji, Zexia Zhang, Zhichao Cui, MeiHua Su

**Affiliations:** ^1^ Department of Rehabilitation Medicine, Dongying People’s Hospital (Dongying Hospital of Shandong Provincial Hospital Group), Dongying, Shandong, China; ^2^ School of Physical Education, Jimei University, Xiamen, Fujian, China

**Keywords:** neuroticism, osteoarthritis, Mendelian randomization, causal relationship, single-nucleotide polymorphisms

## Abstract

**Background:**

Mental health has been found to be associated with risk of osteoarthritis (OA), but the causal relationship was not fully clarified.

**Methods:**

Two-sample Mendelian randomization (MR) study was conducted to investigate the causal relationship between neuroticism (n = 329,821) and the two most frequently affected parts of osteoarthritis (OA) (knee OA: case/control =24,955/378,169; hip OA: case/control = 15,704/378,169) using large scale summary genome-wide association study (GWAS) data. Inverse variance weighted (IVW), weighted median, and MR-Egger were used to estimate the causal effects. Multiple sensitivity analyses were conducted to examine the robustness of the causal estimates. Multivariable MR analysis was used to estimate the direct effects of neuroticism on OA after accounting for the other OA risk factors. Two-step MR approach was employed to explore the potential mediators of the causal relationship.

**Results:**

Univariable MR analysis indicated that 1-SD increase in genetically predicted neuroticism score was associated with an increased risk of knee OA (IVW: OR, 1.17; 95% CI, 1.087–1.26; p = 2.72E−05) but not with hip OA. The causal effects remained significant after accounting for the effects of BMI, alcohol drinking, and vigorous physical activity but were attenuated with adjustment of smoking. Further mediation analysis revealed that smoking initiation mediated a significant proportion of the causal effects of neuroticism on knee OA (proportion of mediation effects in total effects: 22.3%; 95% CI, 5.9%–38.6%; p = 7.60E−03).

**Conclusions:**

Neuroticism has significant causal effects on knee OA risk. Smoking might partly mediate the causal relationship. Further studies were warranted to explore the underlying mechanisms and potential use of neuroticism management for OA treatment.

## 1 Background

Osteoarthritis (OA) is the most common form of degenerative musculoskeletal disorder, and it is generally characterized by degradation of the cartilage within a joint along with the underlying bone remodeling (1). OA can occur in any joints, but knee and hip are the most frequently affected sites. It is estimated that hip and knee OA affects more than 5% of the population worldwide, and this number will continue to rise (2). Age, gender, obesity, and genetic factors have been reported to be associated with the risk of OA (3), but the underlying mechanisms and risk factors for OA have not been fully elucidated.

Accumulating studies have indicated that psychiatric disorders were closely related to the development of OA and pain level (4–7). However, very few studies have explored the relationship between personality traits and OA. Neuroticism, a fundamental and heritable personality trait, is characterized by tendency toward negative emotions such as anxiety, worrying, self-doubt, and loneliness (8). Neuroticism has been found to be closely linked with poor mental health, especially depression (9). Given that the personality traits may influence one’s behavior and biological processes in an earlier and more consistent way compared to the late-onset mental disorders (10), we hypothesized that neuroticism may act as a precursor of psychiatric disorders and thus render a significant impact on OA. A recent observational study found that people with neurotic illnesses or personality disorders were significantly more likely to develop OA during the 7-year follow-up period (4). However, the observational studies are limited to dissecting the causal relationships due to the confounding effects and reverse causality bias (11).

Mendelian randomization (MR) was a recently developed approach using genetic variants as proxy to infer causal relationship between exposures and outcomes (12). Because the genetic variants were randomly distributed and fixed in the process of mitosis, the bias of confounding factors and reverse causation would be effectively minimized (13). MR has been used to explore the causal effects of neuroticism on multiple illnesses, such as depression and stroke (14, 15), but the causal relationship between neuroticism and OA remains unclear. In this study, we employed a two-sample MR approach to dissect the causal relationship between neuroticism and OA (both knee and hip OA). Multivariable MR (MVMR) analysis was performed to evaluate whether the causal effects of neuroticism on OA were independent of other risk factors for OA. Moreover, we explored the potential mediators that may mediate the causal relationship.

## 2 Methods

### 2.1 Study design

**Figure 1:**
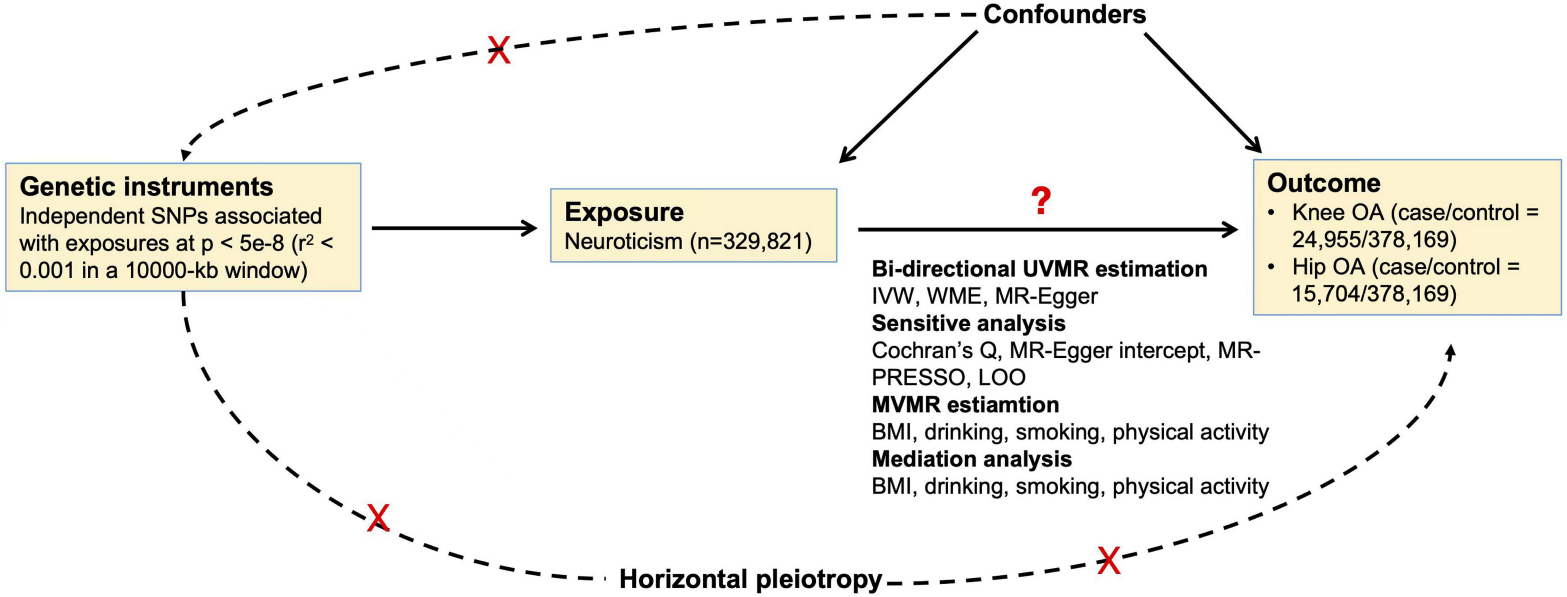
Overview of the study.

The causal relationships between neuroticism and OA were assessed using a two-sample MR design. Three fundamental assumptions should be met for a valid MR estimate: 1. the genetic instruments are robustly associated with the exposure; 2. the genetic instruments were independent of the confounding factors; and 3. the genetic instruments affect the outcome only through the exposure, not via any other alternative factors, i.e., horizontal pleiotropy is not existed (16). The overview of the study can be found in **Figure 1**.

### 2.2 Data sources

The summary statistics for genetic associations with neuroticism were extracted from a large-scale genome-wide association study (GWAS) consisting of 329,821 white British adults from the UK Biobank (UKBB) project (17). Neuroticism was calculated by the sum score of 12-item Eysenck Personality Questionnaire–Revised Short Form (EPQ-R-S) (18). Age, sex, batch number, and genetic principal components were adjusted for the GWAS analysis.

Similarly, we extracted the genetic association data for knee (case/control = 24,955/378,169) and hip OA (case/control = 15,704/378,169) from a large-scale GWAS meta-analysis of two cohorts of UK-based individuals of European ancestry (19). OA was hospital diagnosed or ascertained by the requirement of joint replacement or radiographic evidence. Sex, age, chip, and the top 10 genetic principal components were adjusted for the GWAS study.

As all the summary-level data used for MR analysis are publicly available, there is no need to seek further ethical approval. The detailed information on the study design and ethical approval of the GWAS studies can be found in the original studies.

### 2.3 Genetic instrument selection

Single-nucleotide polymorphisms (SNPs) that are significantly associated with exposures (p< 5E−8) were extracted. Then, the independent genetic instruments were identified by linkage disequilibrium (LD) clumping with the threshold of R^2^< 0.001 in a 10,000-kb window based on the European reference of the 1000 Genomes Project (20). The strengths of the instruments were measured by the F statistics with the formula of $\frac{\left(n-k-1\right)}{k}\times \frac{R^{2}}{1-R^{2}}$ , where n is the sample size, k is the number of SNPs in the instruments, and R^2^ is the proportion of variance explained by the genetic instruments. We ensured that all the instruments used in the MR analysis are larger than 10 to minimize the weak instrument bias (21).

### 2.4 Univariable MR analysis

The random-effect inverse-variance weighted (IVW) was used as the primary method for the causal estimates (21). As IVW is prone to the horizontal pleiotropy bias, we also used two alternative MR approaches (weighted median and MR-Egger) to estimate causal effects. Weighted median would provide an unbiased estimate as long as more than half of the SNPs are valid instruments (22). Given the assumption that instruments are independent of potential horizontal pleiotropy, MR-Egger estimates would be unbiased (23).

Moreover, multiple sensitivity analyses were conducted to ensure that the MR results were robust. Cochran’s Q statistics was used to test the heterogeneity. Two methods (MR-Egger intercept test and MR-PRESSO global test) were used to assess the existence of potential horizontal pleiotropy (23, 24). If significant pleiotropy was detected by the MR-PRESSO global test, we would further test on each of the SNPs to identify potential outliers. The outliers would be removed and the causal effects would be re-estimated using the remaining SNPs. We also conducted the leave-one-out analysis to assess whether the results were dominated by a specific SNP. The causal relationship would be considered significant if a. IVW p-value is less than the Bonferroni-corrected threshold (p< 0.05/2 = 0.025); b. the directions of the three MR estimates were consistent; and c. there is no evidence of significant horizontal pleiotropy identified by MR-Egger intercept test and MR-PRESSO global test (p-values of both methods > 0.05).

### 2.5 Multivariable MR analysis

For the significant causal association identified by the univariable MR analysis, we further performed an MVMR analysis to evaluate the direct effects of neuroticism on OA with adjustment of other known risk factors including body mass index (25), alcohol drinking (26), smoking (27), and vigorous physical activity (28). The direct causal effects were estimated by the multivariable extension of the IVW method (29).

#### 2.5.1 Mediation analysis

To explore the potential mediators of the significant causal relationship, we performed the mediation analysis through the two-step MR approach. In the first step, we estimated the causal effects of neuroticism on the mediator (β1), and, in the second step, the causal effects of mediator on OA were estimated (β2). Then, the mediating effects were calculated as (β1*β2). The significance of the mediating effects and the proportion of the mediating effects in the total effects were estimated by the delta method. Body mass index, alcohol drinking, smoking, and vigorous physical activity were explored as the potential mediators.

All the MR analyses were conducted using the “TwoSampleMR” R package (v.0.5.7) with R (v.4.0.3) (30).

## 3 Results

### 3.1 Genetic instrument selection

After the genetic instrument selection procedures and harmonizing with the outcome data, 60 and 64 SNPs were used for the MR analysis with knee and hip OA respectively (**Supplementary Table S1**). The F statistics are 40.4 and 39.4 for the MR analysis with knee and hip OA, respectively, both of which are larger than the empirical threshold of 10 to minimize the weak instrument bias (21).

### 3.2 Bi-directional univariable MR analysis to explore the causal relationship between neuroticism and OA

**Figure 2:**
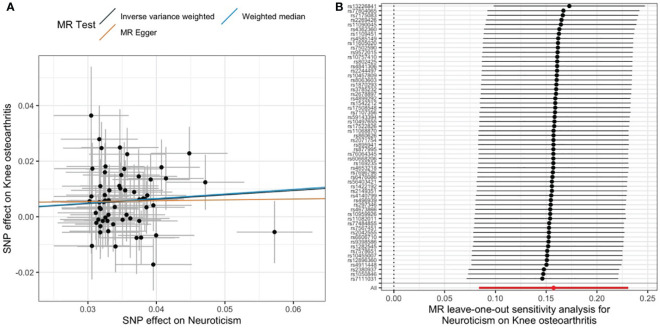
Causal relationship between neuroticism and knee OA. **(A)** Scatter plot of multiple MR estimates. **(B)** Leave-one-out analysis.

We found that 1-SD increase in genetically predicted neuroticism was significantly associated with an increased risk of developing knee OA (IVW: OR, 1.17; 95% CI, 1.087–1.26; p = 2.72E−05). The MR estimates by the methods of weighted median and MR-Egger were comparable (**Figure 2A**). No significant heterogeneity was detected by the Cochran’s Q statistics (p = 0.61). Both MR-Egger intercept (p = 0.669) and MR-PRESSO global test (p = 0.616) did not detect significant horizontal pleiotropy (**Table 1**). Furthermore, the leave-one-out analysis suggested that the causal effects were not driven by a specific SNP (**Figure 2B**). To explore whether knee OA has causal effects on neuroticism, we also conducted the reversal MR with knee OA as the exposure and neuroticism as the outcome. Our results suggested that knee OA has no causal effects on neuroticism (**Supplementary Table S2**).

**Table 1:** The causal effects of neuroticism on osteoarthritis (knee and hip) by univariable MR analysis.

Outcome	nSNP	F_stat	Method	OR (95% CI)	P	Heterogeneity P	MR-Egger intercept P	MR-PRESSO global P
Knee osteoarthritis	60	40.4	IVW	1.17 (1.087–1.26)	2.72E−05	0.61	0.669	0.616
			Weighted median	1.179 (1.061–1.309)	2.12E−03			
			MR Egger	1.031 (0.575–1.848)	9.18E−01			
Hip osteoarthritis	64	39.4	IVW	0.975 (0.879–1.082)	6.38E−01	0.052	0.495	0.045
			Weighted median	1.009 (0.88–1.156)	9.00E−01			
			MR Egger	0.69 (0.255–1.867)	4.67E−01			

IVW, inverse variance weighted; OR, odds ratio.

As for the causal relationship between neuroticism and hip OA, we did not find any significant causal effects in either direction (**Table 1** and **Supplementary Table S2**). In specific, genetically predicted neuroticism was not associated with hip OA (IVW: p = 0.638), whereas genetic proxy for OA was not associated with neuroticism either (IVW: p = 0.78).

### 3.3 Direct causal effects of neuroticism on knee OA by multivariable MR analysis

To further investigate whether neuroticism exerted direct causal effects on knee OA, we performed an MVMR analysis with the adjustment of other risk factors for OA. We found that the significant causal relationship between neuroticism and knee OA remained significant after controlling for the effects of body mass index, alcohol drinking, and vigorous physical activity, but the significance was attenuated with the adjustment of smoking initiation, suggesting that smoking might partly mediate the causal effects (**Table 2**).

**Table 2:** Multivariable MR analysis of the causal effects of neuroticism on knee osteoarthritis with adjustment of other risk factors.

Outcome	Adjustment	OR (95% CI)	P
Knee osteoarthritis	Body mass index	1.161 (1.039–1.298)	8.55E−03
	Alcoholic drinks per week	1.114 (1.001–1.24)	4.87E−02
	Vigorous physical activity	1.115 (1.011–1.23)	2.95E−02
	Smoking initiation	1.078 (0.973–1.194)	1.50E−01

### 3.4 Smoking might partly mediate the causal effects of neuroticism on knee OA

We conducted a mediation analysis using the two-step MR approach to investigate whether the common risk factors for OA mediated the causal pathway between neuroticism and knee OA. Interestingly, we found that smoking initiation might partly mediate the causal effects of neuroticism on knee OA. The proportion of meditation effects in the total effects is estimated to be 22.3% (95% CI, 5.9%–38.6%; p = 7.60E−03). We did not found significant mediating effects of other risk factors including BMI, alcohol drinking, and vigorous physical activity (**Table 3**).

**Table 3:** Mediation analysis of the potential mediators of the causal relationship between neuroticism and knee osteoarthritis.

Outcome	Mediator	Proportion % (95% CI)	P
Knee osteoarthritis	Smoking initiation	22.3 (5.9–38.6)	7.60E−03
	Body mass index	−20.8 (−42–0.5)	5.54E−02
	Alcoholic drinks per week	2.3 (−5.2–9.8)	5.51E−01
	Vigorous physical activity	4.4 (−6.8–15.6)	4.40E−01

## 4 Discussions

In this two-sample MR study, we investigated the causal relationship between neuroticism and OA using the summary data of large-scale GWAS studies. We found that genetically determined neuroticism was significantly associated with knee OA but not with hip OA. The significant causal relationship remained significant after accounting for the effects of BMI, drinking, and physical activity, but the effects were attenuated with the adjustment of smoking initiation. Our further analysis suggested that smoking might partly mediate the causal effects of neuroticism on knee OA. To our best knowledge, this is the first MR study to dissect the causal relationship between neuroticism and OA. Our findings would provide insights into better understanding of the disease pathogenesis, early intervention, and novel treatment development.

Mental health has been increasingly recognized to be associated with the development and the severity of OA (5–7). Most of the current studies focused on the relationship between mental disorders especially depression and OA; however, the impact of personality traits on OA remained largely unknown. Neuroticism is a fundamental personality trait that often being considered as the trigger of the development of mental illnesses especially depression (31). Our analysis revealed that genetically predicted neuroticism leaded to an increased risk of knee OA. This is consistent with a recent finding indicating that people with neurotic illnesses or personality disorders were more likely to develop OA in a prospective cohort study (4). Another study demonstrated that mood disorder was not only associated with risk of OA but also highly correlated with OA pain levels (7). Our MR analysis helped to establish the causal relationship between neuroticism and OA while being less prone to the confounding effects and reverse causality in the observational studies.

The underlying mechanisms through which neuroticism leads to OA were largely unknown. Our mediation analysis indicated that smoking might be a key mediator that links neuroticism and OA. Neuroticism has been reported to be associated with increased risk of cigarette use and persistently daily smoking (32), whereas multiple studies have shown that smoking is significantly associated with OA and pain level (27). Therefore, it is presumed that neuroticism impacted the risk of OA through influencing people’s behaviors such as smoking. Further studies are warranted to investigate the impact of neuroticism associated behavior change on the development of OA.

Another hypothesis is that immune dysregulation might be a shared process of both neuroticism and OA. OA has been widely accepted as an inflammatory disease, in which synovitis and joint inflammation have been found during the pathogenesis of OA. Inflammatory mediators, such as Interleukin-1 (IL-1), interleukin-6 (IL-6), interleukin-15 (IL-15), and tumor necrosis factor-α (TNF-α), have been complicated in OA onset and progression (33). On the other hand, the involvement of inflammation in psychiatric disorders has been increasingly recognized. Neuroticism was found to increase the stress level, and thus lead to upregulation of pro-inflammatory molecules such as IL-1, IL-6, and TNF-α (34). Therefore, the elevated inflammation might position the high neuroticism people at high risk of developing OA. Further investigations were encouraged to study the shared immune pathways in both conditions.

Dissecting the causal relationship between neuroticism and OA has significant clinical implications. As personality traits like neuroticism tend to be early-onset and impact people’s behavior and biological process consistently, early intervention might have profound impact on reducing the risk of developing subsequent disorders like OA. Mindfulness exercise like Yoga and behavior change such as smoking cessation might be able to restore the neuroticism related OA. Further clinical studies were warranted to investigate the efficacy of these non-pharmacological procedures in the prevention and treatment of OA.

There are several limitations of the present study that should not be ignored. First, we restricted the GWAS data to be European participants to avoid the bias due to population stratification. Thus, the findings should not be extended to other ethical groups without further validation using genetically diverse data. Second, the MR approach is inherently impossible to be immune from horizontal pleiotropy; thus, the results might be biased by the unidentified pleiotropic effects. However, multiple MR approaches and sensitivity analyses have yielded consistent and robust results, which would minimize the potential bias by the horizontal pleiotropy. Moreover, we focused on knee and hip OA for the MR analysis because knee and hip are the two most frequently affected parts and have the most available GWAS data. Further studies were warranted to investigate the causal effects of neuroticism on other parts of OA (35). Finally, we focused on the sum neuroticism score as the major exposure in the MR analysis, it would be interesting to explore the causal effects of the 12 sub-items of neuroticism score and other personality traits on the risk of OA in the future studies.

## 5 Conclusion

In conclusion, two-sample MR analysis indicated a significant causal relationship between genetically predicted neuroticism and increased risk of knee OA. Smoking was found to partly mediate this causal relationship. Our findings would provide insights into better understanding of the disease pathogenesis, early intervention, and novel treatment development.

## Data availability statement

The original contributions presented in the study are included in the article/**Supplementary Material**. Further inquiries can be directed to the corresponding author.

## Author contributions

SZ: Writing – original draft, Writing – review & editing. JJ: Writing – original draft, Writing – review & editing. ZZ: Data curation, Writing – original draft. ZC: Data curation, Writing – original draft. MS: Writing – original draft, Writing – review & editing.

## Supplementary material

Supplementary Table 1 The genetics instruments of neuroticism for the MR analysis with knee and hip OA.

Supplementary Table 2 The causal effects of osteoarthritis on neuroticism IVW: Inverse variance weighted; OR: odds ratio.
